# Analysis of Urinary Glycosaminoglycans to Predict Outcome in COVID-19 and Community-Acquired Pneumonia—A Proof-of-Concept Study

**DOI:** 10.3390/jcm12165269

**Published:** 2023-08-13

**Authors:** Alexandros Rovas, Julia Katharina Neumann, Carolin Christina Drost, Richard Vollenberg, Gerold Thölking, Manfred Fobker, Martin Witzenrath, Philipp Kümpers, AGAMOTTO Study Group, CAPNETZ Study Group

**Affiliations:** 1 Department of Medicine D, Division of General Internal and Emergency Medicine, Nephrology, and Rheumatology, University Hospital Muenster, 48149 Muenster, Germany; julia.k.neumann@uni-muenster.de (J.K.N.); carolinchristina.drost@ukmuenster.de (C.C.D.); philipp.kuempers@ukmuenster.de (P.K.); 2 Department of Medicine B, Division of Gastroenterology, Hepatology, Endocrinology and Infectiology, University Hospital Münster, 48149 Muenster, Germany; richard.vollenberg@ukmuenster.de; 3 Department of Internal Medicine and Nephrology, Marienhospital Steinfurt, 48565 Steinfurt, Germany; gerold.thoelking@hjk-muenster.de; 4 Center for Laboratory Medicine, University Hospital Münster, 48149 Muenster, Germany; manfred.fobker@ukmuenster.de; 5 Division of Pulmonary Inflammation, Department of Infectious Diseases and Respiratory Medicine, Charité-Universitätsmedizin Berlin, 10117 Berlin, Germany; martin.witzenrath@charite.de; 6 German Center for Lung Research (DZL), 10117 Berlin, Germany

**Keywords:** COVID-19, 1,9-dimethylmethylene blue (DMMB), endothelial glycocalyx, glycosaminoglycans, community-acquired pneumonia, endothelial dysfunction, endotheliopathy

## Abstract

Although coronavirus disease 2019 (COVID-19) is considered a systemic disease associated with vascular inflammation and eventual destruction of the protective endothelial glycocalyx (eGC), biomarkers of eGC damage are not yet available in the clinic. The most prominent components of eGC are sulphated glycosaminoglycans (sGAGs) attached to core proteoglycans. We hypothesised that the amount of sGAG fragments shed in urine (as a surrogate for systemic eGC damage) would correlate with disease severity and outcome. Total urinary sGAG concentration was measured using an in-house optimised 1,9-dimethylmethylene blue (DMMB) assay, which is highly accurate and insensitive to interferences. The median urinary sGAG concentration was significantly higher in 67 hospitalised patients with COVID-19 compared to 72 hospitalised patients with community-acquired pneumonia (CAP). In both groups, urinary sGAG concentrations predicted a combined endpoint (including intubation and death) with an area under the receiver operator characteristic curve of 0.72 (95% CI 0.55–0.88, *p* = 0.01) and 0.70 (95% CI 0.57–0.83, *p* = 0.007), respectively. In conclusion, the inexpensive and easy-to-perform DMMB assay provides a surrogate parameter for eGC damage that may be useful for risk stratification of patients with COVID-19 and CAP.

## 1. Introduction

Coronavirus disease 2019 (COVID-19) caused by severe acute respiratory syndrome coronavirus 2 (SARS-CoV-2) presents as a systemic disease associated with vascular inflammation and marked endothelial injury [1,2]. In addition to COVID-19, microvascular and endothelial dysfunction is also a hallmark of inflammation in sepsis [3].

The vascular endothelium is lined by a gel-like layer of highly sulphated glycosaminoglycans (sGAGs) attached to core proteoglycans, the so-called endothelial glycocalyx (eGC). This fragile, negatively charged structure shields the endothelium from pathogenic insults and plays a key role in the maintenance of microcirculatory homeostasis. Inflammation-induced eGC dysfunction leads to vascular barrier breakdown, hyperpermeability and consequent oedema and organ damage in critically ill, and especially septic, patients [4].

Glycocalyx impairment can currently be assessed either by enzyme-linked immunosorbent assays (ELISA) of eGC constituents in blood samples or in vivo using a novel quantitative sublingual video microscopy technique, the so-called GlycoCheck^TM^ system [3]. Using this video microscopy technique, we have recently shown that damage (thinning) of the endothelial glycocalyx (eGC) predicts in-hospital mortality in COVID-19 patients [5]. Although this finding is promising, widespread clinical use of this sophisticated and expensive technique does not appear feasible at present.

The total concentration of sGAGs in urine can be easily measured using the 1,9-dimethylmethylene blue (DMMB) assay, which is mainly used to screen for mucopolysaccharidosis in paediatrics [6]. Only recently, pilot studies in critically ill patients with septic shock [7] and severe falciparum malaria [8,9] have shown that urinary sGAG fragment levels (as a surrogate for systemic eGC injury) correlate with disease severity and predict outcome.

The aims of this study were (1) to characterise, improve pre-analytics and further develop the DMMB assay and (2) to determine whether urinary sGAG can predict outcome in COVID-19 and community-acquired pneumonia (CAP).

## 2. Materials and Methods

### 2.1. Study Population

#### 2.1.1. COVID-19 Cohort and Healthy Controls

A total of 67 PCR-confirmed adult COVID-19 patients (delta variant), presenting to the Emergency Department (ED) of Münster University Hospital or one of four local teaching hospitals requiring inpatient admission, were prospectively and non-consecutively enrolled between June and August 2021. After written informed consent was obtained, urine samples were collected within 24 h after hospital admission and stored at −20 °C until analysis. Exclusion criteria were underage, palliative end-of-life care, pregnancy and haemodialysis. Ten apparently healthy controls were used to determine the normal range of the DMMB assay. The study was conducted in accordance with the Declaration of Helsinki and approved by the Ethics Committee of the General Medical Council Westfalen-Lippe and the WWU Münster, Germany (file number: amendment to 2020-869-f-S—Approved: 30 December 2020).

#### 2.1.2. Community-Acquired Pneumonia (CAP) Cohort

A total of 72 hospitalised adult patients with confirmed CAP who participated in the CAPNETZ observational study [10] in Germany between January 2014 and April 2017 were included in the study. Urine samples were collected at enrolment and stored at −80 °C until analysis. The CAPNETZ study (German Clinical Trials Register: DRKS00005274) was approved by the Ethics Committee of the Medical Faculty of the Otto-von-Guericke University Magdeburg (file number: 104/01).

### 2.2. Reagents and Consumables

DMMB zinc chloride double salt and Chondroitin sulphate A (CS) sodium salt from bovine trachea were purchased (Sigma-Aldrich, St. Louis, MO, USA). The DMMB reagent was prepared according to a standard protocol, in which DMMB (16 mg), glycine (3.05 g), sodium chloride (1.6 g) and acetic acid (544 μL) were made up to a volume of 1 L with distilled water and stored at 4 °C without exposure to light for up to a month or until precipitation occurred [11,12].

For the assay, 10 µL of the dilution series or urine samples was transferred to a 96-well microplate for duplicate measurements. DMMB solution was brought to room temperature, and then 200 µL was added using an Eppendorf multidispenser. The absorbance was read immediately using a Tecan infinite 200 microplate reader and the i-control™ software (Tecan, 2.0.10.0, Männedorf, Switzerland). For each experiment, a dilution series of CS was prepared to establish a standard curve from 0 to 125 µg/mL.

In further experiments, urine samples were spiked with CS (Sigma-Aldrich, St. Louis, MO, USA), albumin (5 mg/mL), glucose (10 mg/mL), calcium chloride (3 mg/mL) or acetone (7.9 mg/mL). Chondroitinase (Sigma-Aldrich, St. Louis, MO, USA) and deoxyribonuclease I were used for enzymatic digestion of CS or DNA, respectively, in urine samples.

### 2.3. Evaluation and Further Development of DMMB Assay

To evaluate and characterize the DMMB assay, we assessed the following parameters [13]:

- Analytical assay sensitivity, hereafter assay sensitivity, defined as the slope of the calibration curve.
- Limit of Blank (LoB) is the highest apparent analyte concentration expected to be found when replicates of a blank sample containing no analyte are tested. LoB = mean_blank_ + 1.645 × (SD_blank_)
- Limit of Detection (LoD) is defined as the lowest analyte concentration that can be reliably distinguished from the LoB and at which detection is feasible. The LoD is determined by using both the measured LoB and test replicates of a sample known to contain a low concentration of analyte. LoD = LoB + 1.645 × (SD low-concentration sample)
- Intra-assay precision, defined as the within-run precision of the assay and assessed as the coefficient of variation of 8 parallel measurements of four samples (SD × 100/mean) and inter-assay precision.
- Inter-assay precision, assessed by measurement of four samples in different runs by different operators.
- Stability of the urine samples was assessed by evaluating the effect of storage, exposure to light as well as freeze–thaw cycles and centrifugation.

Automated analysis was performed using a COBAS INTEGRA 400 plus laboratory analyser (ROCHE, Mannheim, Germany).

### 2.4. Statistical Analysis

Data are presented as absolute values with means or standard error of the mean (SEM) or median with corresponding 25th and 75th percentiles (interquartile range (IQR)), as appropriate. Differences between groups were tested with non-parametric Mann–Whitney U test and the chi square test. Linear regression analysis was used to assess associations between variables. Receiver operator characteristic (ROC) analysis was used to assess the area under the curve (AUROC) and to establish different sGAG cut-offs. All tests were two-tailed, and significance was set at *p* < 0.05. GraphPad Prism version 8.4.3 (GraphPad Prism Software Inc, San Diego, California, USA) and SPSS 29 (IBM, Armonk, New York, NY, USA) were used for data analysis and figure preparation.

## 3. Results

### 3.1. Sensitivity, Precision and Freeze–Thaw Studies

**Figure 1:**
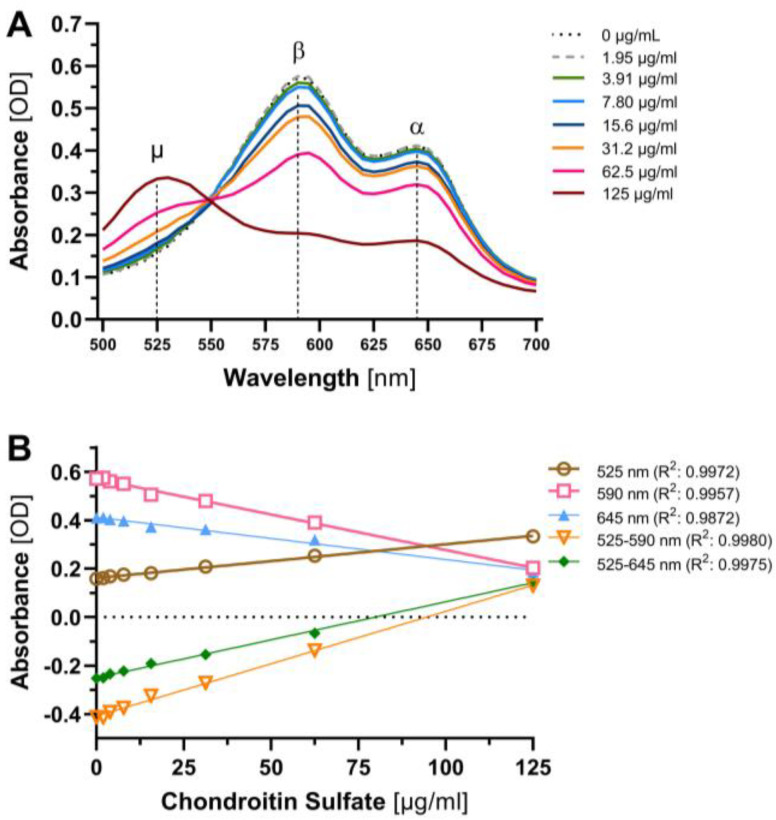
Improving analytical sensitivity by adoption of a wavelength difference method. (**A**) Spectral scan of the 1,9-dimethylmethylene blue (DMMB) assay from 500 to 700 nm in 5 nm steps showing changes in absorbance (optical density (OD)) depending on concentration of sulfated glycosaminoglycans (sGAG). Dotted lines indicate α peak at 645 nm, β peak at 590 nm and μ peak at 525 nm, respectively. (**B**) Assay sensitivity for wavelength 525, 590 and 645 nm as well as wavelength difference 525–590 and 525–645 nm. Higher slope of the standard curve corresponds to higher sensitivity.

Spectral scans performed in increments of 5 nm from 500 nm to 700 nm using a Tecan infinite 200 microplate reader and the i-control™ software (Tecan, 2.0.10.0, Männedorf, Switzerland) showed that increasing CS concentrations resulted in a decrease in the β (590 nm) and α (645 nm) peaks and an increase in the μ (525 nm) peak (Figure 1A). The assay sensitivity, defined as the slope of the standard curve, was highest at 590 nm (110% higher than at 525 or 68% higher than at 645 nm) and at pH 4 (~20% higher than at pH 3 or 5). The use of a wavelength difference method (μ minus β or α peak [14]) significantly enhanced the sensitivity (i.e., the slope of the regression curve) to CS by 163% (525–590 nm) or 91% (525–645 nm), respectively, compared with measurements at the single wavelength of 590 nm (Figure 1B). Therefore, the 525–590 nm difference at pH 4 was used as the standard for further experiments.

The limit of blank of the assay (LoB = mean_blank_ + 1.645 × (SD_blank_)) was 2.25 µg/mL, and the limit of detection (LoD = LoB + 1.645 × (SD_low concentration sample_)) was 5.08 µg/mL [13]. Urine samples from patients with septic shock (considered a positive control) and healthy subjects (*n* = 4 per group) were used to further characterise the assay. The within-run (intra-assay) precision, assessed as the coefficient of variation (SD × 100 / mean) of 8 parallel measurements of four samples, was 1.7% for septic shock and 11.7% for healthy subjects. The inter-assay precision (4 samples measured on different runs by different operators) was 7.5%. Urinary sGAG values were stable for at least 24 h when stored at room temperature (median (IQR)) 96% (92% to 101%) vs. 100% at baseline), were unaffected by exposure to light (97% (91% to 112%)) and showed only a slight decrease (91% (78% to 113%)) after four cycles of thawing (5 h) and refreezing (19 h).

### 3.2. Interference, Specificity and Centrifugation Studies

**Figure 2:**
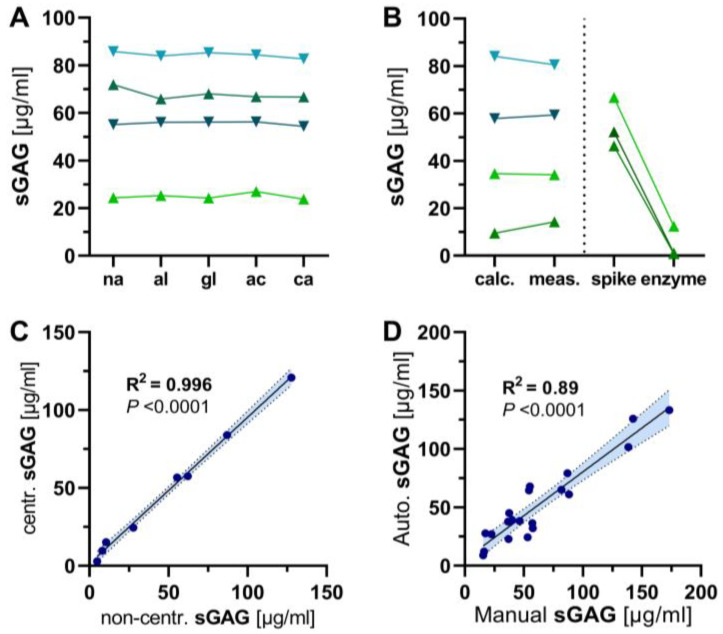
Pre-analytics of the DMMB assay using urine samples. (**A**–**D**): DMMB assay characterization with urinary samples from septic shock patients and healthy controls. Same samples are coded by same colours. (**A**) Influence of possible interfering urinary constituents. Native samples (na) of septic shock patients or healthy controls (*n* = 2 each) were spiked with supra-physiologic concentrations of albumin (al), glucose (gl), acetone (ac) or calcium chloride (ca). (**B**) Samples were spiked with 62.5 µg/mL Chondroitinsulfate (CS) solution. Calculated concentrations (calc.) of sulfated glycosaminoglycans (sGAG) were compared to concentrations measured with DMMB assay (meas.). Urine samples from healthy subjects (*n* = 3) were spiked with 250 µg/mL CS solution (spike) and subsequently digested with chondroitinase (enzyme). (**C**,**D**) Linear regression with 95% confidence interval between (**C**) centrifugated and non-centrifugated samples, as well as (**D**) manual and semi-automatic-operated DMMB assay.

The addition of albumin (5 mg/mL), glucose (10 mg/mL), calcium chloride (3 mg/mL) or acetone (7.9 mg/mL) to simulate significant albuminuria, glucosuria, hypercalciuria or ketonuria resulted in no significant change in the measured urinary sGAG concentration (Figure 2A). There was good agreement between expected and measured sGAG levels in urine samples spiked with an additional 62.5 µg/mL CS solution. The addition of chondroitinase (Sigma-Aldrich, St. Louis, MO, USA ) to urine samples spiked with 250 µg/mL CS solution resulted in almost complete enzymatic degradation of spiked CS (Figure 2B). In contrast, the addition of deoxyribonuclease I did not alter urinary sGAG concentrations, indicating that interference from DNA [14] is negligible in urine samples. Centrifugation of the samples had no effect (Figure 2C).

We also evaluated its amenability to fully automated processing and detection using the COBAS INTEGRA 400 plus laboratory analyser (ROCHE, Mannheim, Germany). The LoB and LoD determined for the automated method were 1.23 µg/mL and 5.41 µg/mL, respectively. The intraassay precisions were 9.5% and 8.2% (*n* = 20) at low and high analyte concentrations, respectively, and intra-assay accuracies were 3.4% and 2.7% (*n* = 20) at low and high analyte concentrations, respectively. The agreement between the two methods was very good (R² = 0.89, *p* < 0.0001) (Figure 2D).

### 3.3. Baseline Characteristics

COVID-19 patients were 62 (50–74) years old, often had comorbidities and 33 (49.3%) required oxygen supplementation at the time of urine collection. Acute respiratory distress syndrome (ARDS, Berlin definition) occurred in 15 (22.4%) patients. Of these, seven (10.4%) required non-invasive ventilation (NIV) or high-flow nasal cannula oxygen therapy but not invasive ventilation, and eight (11.9%) required intubation for invasive ventilation (Table 1).

Patients with CAP were 78 (70–85) years old and often had pre-existing lung disease (41.7%). Of these, two (2.8%) required NIV followed by invasive ventilation (Table 1).

### 3.4. Urinary sGAG Concentrations in COVID-19 and CAP

**Figure 3:**
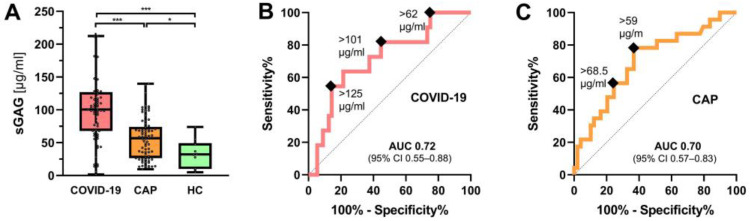
Clinical application of the DMMB assay in COVID-19 and CAP. (**A**) Boxplots showing urinary sGAG concentration COVID-19 patients (*n* = 67) and patients with community-acquired pneumonia (CAP, *n* = 72). Healthy controls (HC, *n* = 10) served to establish reference values. Groups were compared by the Mann–Whitney test (* *p* < 0.05, *** *p* < 0.0001). Receiver-operator characteristic (ROC) curve analysis of sGAG concentration for prediction of the combined outcome in (**B**) COVID-19 patients (moderate-to-severe ARDS, intubation, or death) and (**C**) CAP (intubation or death).

The median urinary sGAG concentration was significantly higher in COVID-19 patients compared to CAP patients (101 (68–212) µg/mL vs. 57 (26–140) µg/mL, *p* < 0.001). The median sGAG concentrations of both groups were significantly above the normal range found in healthy subjects (Figure 3A).

In the COVID-19 cohort, a receiver operator characteristic (ROC) curve analysis showed that urinary sGAG concentration (525–590 nm method) predicted a combined endpoint (moderate-to-severe ARDS, intubation or death, *n* = 11) with an area under the receiver operator characteristic curve (AUROC) of 0.72 (95% CI 0.55–0.88), *p* = 0.01 (Figure 3B). The association between urinary sGAG levels and the combined outcome remained significant after adjustment for age, sex, quick sequential organ failure assessment (qSOFA) score and C-reactive protein levels in a regression model: hazard rate (HR) = 1.025 (95% CI 1.003–1.047), *p* = 0.023. Neither ferritin nor interleukin-6 or D-dimers showed any predictive value for the combined endpoint (all *p* > 0.05).

In CAP, urinary sGAG concentration also predicted the combined endpoint (intubation or death, *n* = 23) with an AUROC of 0.70 (95% CI 0.57–0.83), *p* = 0.007 (Figure 3C). This association between urinary sGAG values and the combined outcome remained significant after adjustment for age, sex, qSOFA and C-reactive protein (HR = 1.018 (95% CI 1.001–1.036), *p* = 0.041).

## 4. Discussion

In this study, we comprehensively characterized the stability and the pre-analytics of the DMMB assay for the determination of sGAG in the urine of critically ill patients. The assay showed good inter- and intra-assay precision and was not susceptible to interference from common urinary substances. The measurement of sGAG in urine was stable, even at room temperature, and was not affected by freeze–thaw cycles or centrifugation. Furthermore, the procedure could be automated in our laboratory with a good agreement between the hand-operated and automatic method. The combination of different wavelengths allowed us to further improve the analytical assay sensitivity. All these characteristics together demonstrate the suitability of this colorimetric assay as a reliable and robust method for the determination of sGAG in the urine, with great potential for development as a point-of-care test at the patient’s bedside.

In the clinical setting of COVID-19 and CAP, we found that urinary sGAG levels were significantly higher on hospital admission compared to healthy controls. Urinary sGAG levels were similarly predictive of the combined outcome in both groups. This result confirms and extends previous findings in critically ill patients with septic shock, ARDS and malaria [7,9]. However, it is noteworthy that the sGAG levels in COVID-19 patients were, on average, twice as high as those of CAP patients, despite the latter having a three-fold higher in-hospital mortality. The comparison between the two groups should be treated with caution, as they were not well matched for age, gender and disease severity, and this was also not the aim of our study. However, at an explorative second glance, it fits well with the pathophysiological concept of CAP as being primarily a local inflammation of the lungs with, in some cases, secondary systemic spread, whereas even moderate COVID-19 appears to induce pronounced endothelial inflammatory responses [5,15]. The differences between the two groups currently preclude the establishment of a single “cut-off” value. Interestingly, previous studies comparing urinary sGAG in patients with sepsis-induced ARDS and pneumonia also showed a markedly increased sGAG concentration in the ARDS group [7]. Indeed, ARDS is associated with overt glycocalyx damage and higher plasma GAG concentrations [16]. Therefore, the higher prevalence of ARDS in COVID-19 may also partially explain the observed results.

Measurement of urinary sGAG directly at hospital admission could be an easy-to-perform risk stratification tool and provide a robust alternative to sophisticated glycocalyx measurements. Data from animal studies suggest that damage to the glycocalyx occurs very early in systemic inflammation [16]. It is, therefore, tempting to speculate that urinary sGAG may serve as an early indicator of inflammatory endothelial involvement in COVID-19 and CAP. We have recently shown that the proteomic signature of a destroyed glycocalyx is, indeed, a biomarker that correlates with disease severity and predicts 28-day mortality and/or intubation in an independent COVID-19 cohort [17]. Further studies may investigate whether this also applies to other pulmonary and non-pulmonary inflammatory diseases with vascular involvement.

Our study has some limitations that need to be addressed. First, the sample size of both cohorts studied here was limited. However, both cohorts were recruited in a multicentre, prospective manner, each of which included patients who required hospitalization. As the urine samples were collected on admission, the design of our study differs significantly from many cross-sectional studies in intensive care unit patients, who are already at high risk of poor outcome. Second, we did not record whether the patients had already received (low-molecular-weight) heparin (LMWH) at the time of study enrolment. Although only about 3–6% of these are excreted renally [18], we cannot completely rule out an influence on measured sGAG levels. However, one would expect the proportion of prophylactic heparin/LMWH administration to be similar in both cohorts. Third, we cannot exclude that the longer storage time could have contributed to the lower sGAG values in CAP. Fourth, urinary sGAG concentration has been reported to be influenced by the menstrual cycle or hormonal preparations in females [19,20]. However, we believe that this is negligible in our study as no difference was observed after adjustment for sex. Fifth, as we did not simultaneously measure sublingual glycocalyx thickness or circulating glycocalyx components in the blood in this study, we cannot make any statements about the congruence or superiority of the different measurement methods. Pilot studies have shown that the core protein syndecan-1 and heparan sulphate levels are elevated in COVID-19, although the predictive power increases with disease severity or during disease progression [21,22,23]. Compared to the analysis of syndecan-1 or heparan sulphate in plasma, the determination of sGAG in urine appears to be less specific at first glance. However, since the DMMB assay measures all sulphated GAG types (heparan sulphate, chondroitin sulphate, dermatan sulphate and keratan sulphate) at the same time, damage to the eGC is probably detected very sensitively. Further longitudinal studies, ideally with accompanying blood sampling and sublingual video-microscopy, are needed to confirm this hypothesis.

## 5. Conclusions

Our proof-of-concept study demonstrates that measurement of sGAG in urine can serve as a robust, inexpensive (~ EUR 1 per sample) and easy-to-perform test to measure glycocalyx damage in COVID-19 and CAP. Implementation of this simple and inexpensive assay in routine clinical practice could help to focus our attention on vascular damage, which is often neglected. Whether urinary sGAG has additional value to other established biomarkers for the prediction of clinical endpoints needs to be clarified in larger studies.

## Figures and Tables

**Table 1:** Baseline characteristics.

Variable	COVID-19 Cohort	CAP Cohort *
**Baseline characteristics**		
Number of participants (*n*; %)	67 (100)	72 (100)
Female sex (*n*; %)	36 (53.7)	22 (30.6)
Age (years, median (IQR))	62 (50–74)	78 (70–85)
BMI (kg/m^2^, median (IQR))	27 (24–31)	na
Positive SARS-CoV-2 swap * (*n*; %)	67 (100)	na
Quick SOFA score (pts, median (IQR)	0 (0–2)	0 (0–2)
Oxygen supply (*n*; %)	33 (49.3)	50 (69.4)
C-reactive protein (mg/dl median (IQR))	4.6 (1.5–4.2)	7.4 (3.1–15.2)
**Comorbidities** (*n*; %)		
Cardiovascular disease	40 (59.7)	20 (27.8)
Kidney disease	11 (16.4)	9 (12.5)
Lung disease	14 (20.9)	30 (41.7)
Diabetes	19 (28.4)	na
Malignancy	1 (1.5)	10 (13.9)
**Outcomes** (*n*; %)		
ARDS	15 (22.4)	na
Intubation	8 (11.9)	2 (2.8)
NIV or HFNC	7 (10.4)	2 (2.8)
In-hospital mortality	8 (11.9)	23 (31.9)

* The CAP cohort was enrolled from 2014 to 2017, i.e., before the COVID-19 pandemic. Abbreviations: CAP = community-acquired pneumonia, COVID-19 = Coronavirus disease 2019, BMI = Body mass index, na = not applicable, SOFA score = Sequential Organ Failure Assessment score, ARDS = acute respiratory distress syndrome, NIV = non-invasive ventilation, HFNC = high-flow nasal canula oxygen therapy.

## Data Availability

The datasets used and/or analyses during the current study are available from the corresponding author on reasonable request.
